# 

**DOI:** 10.3201/eid0908.030041

**Published:** 2003-08

**Authors:** Hermann Feldmeier, Margit Eisele, Rômulo César Sabóia-Moura, Jörg Heukelbach

**Affiliations:** * Free University of Berlin, Berlin, Germany; † Mandacaru Foundation, Fortaleza, Brazil

**Keywords:** tungiasis, *Tunga penetrans*, pathology, severe morbidity, Brazil, research

## Abstract

Tungiasis is caused by infestation with the sand flea (*Tunga penetrans*). This ectoparasitosis is endemic in economically depressed communities in South American and African countries. Tungiasis is usually considered an entomologic nuisance and does not receive much attention from healthcare professionals. During a study on tungiasis-related disease in an economically depressed area in Fortaleza, northeast Brazil, we identified 16 persons infested with an extremely high number of parasites. These patients had >50 lesions each and showed signs of intense acute and chronic inflammation. Superinfection of the lesions had led to pustule formation, suppuration, and ulceration. Debilitating sequelae, such as loss of nails and difficulty in walking, were constant. In economically depressed urban neighborhoods characterized by a high transmission potential, poor housing conditions, social neglect, and inadequate healthcare behavior, tungiasis may develop into severe disease.

Tungiasis is a common, but neglected, health problem in economically depressed communities in South American and sub-Saharan African countries (*1*–*5*). This ectoparasitosis is caused by the sand flea (*Tunga penetrans,* Siphonaptera: Tungidae, Tunginae), also called the jigger flea. The female jigger flea penetrates into the skin of its host, undergoes a peculiar hypertrophy, expels several hundred eggs for a period of <3 weeks, and eventually dies. The shriveled carcass is then sloughed from the epidermis by host repair mechanisms (*6*,*7*). Within 10 days, the flea increases its volume by a factor of approximately 2,000, finally reaching the size of a pea. Through its hindquarters, which serve for breathing, defecating, and expulsing eggs, the flea remains in contact with the air, leaving a sore (240–500 μm) in the skin; the sore is an entry point for pathogenic microorganisms (*8*). The preferred localization for jiggers is the periungual region of the toes, but lesions may occur on any part of the body (*9*).

Tungiasis, a zoonosis, affects a broad range of domestic and peridomestic animals, such as dogs, cats, pigs, and rats (*10*). Where humans live in close contact with these animals and where environmental factors and human behavior favor exposure, the risk for infection is high (*3*,*11*).

Numerous case reports detail the clinical aspects of tungiasis. However, they almost all exclusively describe travelers who have returned from the tropics with a mild disease (*12*). Having reviewed 14 cases of tungiasis imported to the United States, Sanushi (*13*) reported that the patients showed only one or two lesions and, that except for itching and local pain, no clinical pathology was observed. In contrast, older observations show that indigenous populations and recent immigrants, as well as deployed military personnel, frequently suffered from severe disease, characterized by deep ulcerations, tissue necrosis leading to denudation of bones, and auto-amputation of digits, resulting in physical disability, such as being unable to work and walk (*14*–*19*). Tungiasis has also been associated with lethal tetanus in nonvaccinated persons (*19*–*22*). In a study in São Paulo State, Brazil, tungiasis was identified as the place of entry in 10% of tetanus cases (*23*).

We present the clinical findings as well as the demographic and environmental characteristics of 16 persons with severe tungiasis who were identified during a prospective study on *Tunga penetrans–*associated disease at a Primary Health Care Center (PHCC) in a economically depressed neighborhood (*favela*) in Fortaleza, northeast Brazil. The results indicate that in resource-poor populations important disease may frequently occur and seems to be related to a combination of intense transmission, economic deprivation, social neglect, and inadequate healthcare behavior.

## Materials and Methods

### Study Area

The *favela* Morro de Sandras is on the outskirts of Fortaleza, the capital of Ceará State, northeast Brazil, and is similar to other economically depressed areas there. During the high transmission season (July–December), approximately one third of the population is affected by tungiasis (*24*). Other ectoparasitic diseases such as head lice, scabies, and cutaneous larva migrans are also very common in the study area (*25*). This area is built on a dune close to the beach and has a total population of 1,500 persons. Sixty percent of the population has a monthly family income of less than two minimum wages (1 minimum wage = US$80.00). Adult illiteracy is 30%, unemployment rates are high, and crime is common. Ninety-seven percent of the households have electricity, and about 60% have access to running water (*26*). Many houses are made with improvised construction material and do not have concrete floors. Waste and sewage disposal are insufficient, and hygienic conditions are precarious. Most streets are not paved. Innumerable stray dogs and cats roam the area, in addition to dogs and cats kept as pets. Rodents are numerous; *Rattus rattus* can be seen during the day feeding on organic waste disposed of in backyards or outside family compounds. The prevalence of tungiasis ranges from 5% to 35% according to the season (J. Heukelbach, unpub. data).

### Study Population

The study was performed at the PHCC that serves the population of the *favela*. During a 6-week period, 86 persons with tungiasis were identified among patients who visited the center for medical reasons unrelated to the ectoparasitosis. Severe tungiasis was arbitrarily defined as the presence of >50 lesions. Sixteen of the 86 patients fulfilled this criterion and are described in this case series. They ranged in age from 2 to 50 years of age.

### Clinical Examination

As tungiasis may occur at any topographic site (*9*), the whole body surface of the patient was examined for the presence of vital, egg-producing, involuting, or dead fleas. Lesions were classified according to the Fortaleza Classification, a recently elaborated staging system (*7*). The following findings were considered diagnostic for tungiasis: flea in statu penetrandi, stage I, a dark and itching spot in the epidermis with a diameter of 1–2 mm with or without local pain; stage II (early lesion), lesions with as a white halo with a diameter of 3–10 mm with a central black dot; stage III (mature flea), a brownish-black circular crust with or without necrosis of the surrounding epidermis; stage IV (dead parasite), circular residue punched out in the keratin layer of the sole of the foot or irregular thickening of the nail rim; and stage V, lesions altered through manipulation by the patient (such as partially or totally removed fleas, which leaves a characteristic crater-like sore in the skin) and suppurative lesions, mainly caused by using nonsterile perforating instruments such as needles and thorns.

During the examination, location and number of lesions were noted, and the following signs and symptoms were observed: erythema, edema, tenderness, itching, pain, shining skin, desquamation, hyperkeratosis, fissures, pustules, suppuration, ulcers, deformation of the toes (defined as deviation of the normal axis of the toe caused by intense swelling), deformation of nails, loss of nails, and difficulty in walking or gripping.

Clinical pathologic findings were classified as follows: acute inflammation or painful lesion surrounded by erythema, edema, and tenderness; chronic inflammation, edema, tenderness, shining skin with or without desquamation, or deformation of digits; superinfection, presence of pustules, suppuration, or ulcers; and physical disability, difficulty in walking, or gripping (if lesions were located on the hands), based on patients’ statements that pain restricted their movements. Lesions tended to occur in clusters, which were arbitrarily defined as a group of five or more lesions that occurred in close proximity (e.g., on the periungual region of the toe, the heel, or the fingertip).

### Statistical Analysis

Statistical analysis was performed by using the StatView software package version 1.5 (SAS, Cary, NC). The Wilcoxon signed rank test, the Spearman rank correlation coefficient test, and the Fisher exact test were applied when appropriate.

### Ethical Considerations

The study was approved by the Ethical Committee of the Federal University of Ceará State, Fortaleza, Brazil. Before the study, meetings with community health workers, community leaders, and staff members of the PHCC were held in which the objectives of the study were explained. Informed written consent was obtained from each patient after the objectives of the study were explained. In the case of a minor, the caregivers were asked for their consent. After the examination, all patients were treated topically with thiabendazole 5% and, in the case of superinfection, with neomycin ointment. All patients received a pair of tennis shoes and were encouraged not to walk barefoot.

## Results

**Figure 1:**
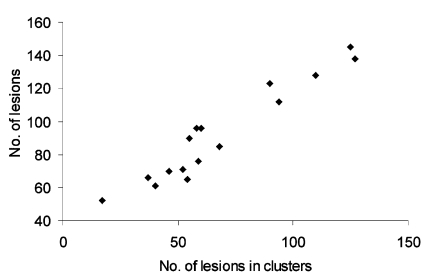
Correlation between total number of lesions and number of lesions occurring in clusters (rho=0.94; p=0.0003).

The demographic characteristics of the patients in the study and the number of lesions present are shown in Table 1. Patients had at least 52 lesions with a maximum of 145 and a median of 88 lesions. Of the 1,474 lesions, 1,092 (76%) occurred in clusters. A significant correlation existed between the number of lesions per patient and the number of lesions occurring in clusters (rho=0.94; p=0.003; Figure 1). No significant relationship existed between the number of lesions and age (rho=0.44, p>0.05). However, manipulated lesions were more frequent in patients >15 years of age (38% vs. 13%; p<0.05). No difference existed in the median number of lesions between female and male patients.

**Table 1:** Demographic and parasitologic characteristics and number of lesions in 16 patients with severe tungiasis

Characteristics	No./total
Median age in y (and range)	12 (2–50)
Female/male	7/9
Median no. of lesions/patient (range)	88 (52–145)
Vital lesions (stages I–III)	43 (10–77)
Dead lesions/residuals (stages IV–V)	38 (11–85)
Manipulated lesions	14 (1–46)
Presence of clusters in individual patients	16/16 (100%)
Lesions occurring in clusters	1,092/1,474 (76%)
Median no. of lesions/cluster (range)	12 (6–30)

The topographic distribution of lesions is summarized in Table 2. All patients had lesions on the toes and soles of the feet. The periungual region of the toe was clearly a predilection site. Other regions of the toes, such as the tip, the plantar, or the dorsal site were also frequently affected. Fifteen patients (88%) had lesions on their heels, and two patients (13%) had lesions on the dorsum pedis. Lesions on the hands were found in six patients (38%); one person had a lesion in the gluteal area, and another patient had a lesion on the chest.

**Table 2:** Distribution of lesions according to topographic site

Topographic site	No. of lesions median (range)	% of all lesions (n=1,474)
Toes	48 (17–101)	56.4
Periungual	29 (9–58)	29.0
Other areas^a^	17 (8–66)	27.4
Sole	20 (3–48)	20.1
Heel	9 (0–62)	20.5
Dorsal area	0 (0–3)	0.3
Hands/gluteal region	0 (0–21)/0 (0–1)	2.7

^a^Such as the tip, the plantar, or the palmar side of the toes.

Clinical findings are shown in Table 3. In all patients, signs of acute as well as chronic inflammation were present. Acute and chronic inflammation occurred simultaneously in different topographic sites or in lesions at different stages of development. In three patients, the entire foot and lower leg were inflamed. Deformation or nail loss was common (69%). All patients had difficulty walking, and half of the patients with lesions at the fingers had difficulty gripping. Intense itching, a common symptom, prevented patients from sleeping soundly. The number of lesions was particularly high in patients with signs of generalized inflammation, when fissures were present or when superinfection had occurred (Table 4). We describe four cases that are typical examples of severe tungiasis.

**Table 3:** Clinical findings in patients with severe tungiasis (n=16)

Clinical pathology observed	Present in patients (%)
Acute local inflammation	16/16 (100)
Chronic local inflammation^a^	16/16 (100)
Generalized inflammation^b^	3/16 (19)
Superinfection	7/16 (31)
Pustule(s)	3/16 (19)
Suppuration	4/16 (25)
Severe itching	16/16 (100)
Ulcers	8/16 (50)
Fissures	3/16 (19)
Deformation or loss of nails	11/16 (69)
Difficulty in walking	16/16 (100)
Difficulty in gripping^c^	3/6 (50)

^a^Edema, desquamation, shining skin, hyperkeratosis with or without deformation of digits.

^b^Edema and pain of entire foot with or without suppuration.

^c^Six patients had lesions on the hands.

**Table 4:** Relationship between number of lesions and selected clinical pathologic findings

Clinical pathology observed	Median no. of lesions
General inflammation	131
Fissures	128
Pustules or suppuration	123
Ulceration	88
Deformation or loss of nails	81

### Case 1

A 2-year-old girl had 90 lesions; 49 of these lesions were located on the toes, 33 on the soles of the feet, and 3 on the heels. Five lesions were at ectopic sites (four of them on the fingertips and one in the gluteal region). One sand flea was trying to penetrate the skin of the chest. Many lesions were superinfected as indicated by pustules or suppuration, and the nails of six toes were deformed or had already fallen off. The lesions on the fingertips were particularly painful and caused difficulty gripping. The child also had pediculosis and was underweight. The family (three children and the parents) lived in a small hut with a sandy floor. The mother was 18 years of age; both parents were illiterate.

### Case 2

**Figure 2:**
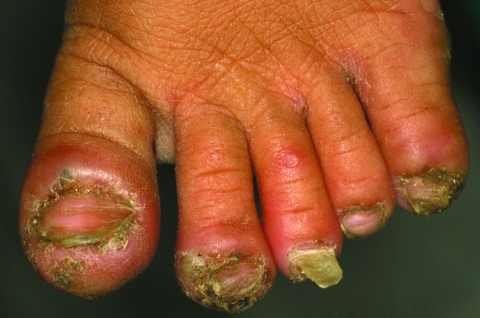
Left foot of a 6-year-old girl. The first, second, third, and fifth toe are infected with *Tunga penetrans*. These toes are inflamed, and the second and the third toe are distorted by severe edema. The first toe shows hyperkeratosis. The nails of the first, second, and fifth toe are deformed, and the nail of the third toe is falling off. A flea is trying to penetrate the skin at the edge of the pustule on the medial side of the second toe (11 clockwise). An ulcer has formed above the proximal phalangeal joint of the third toe.

**Figure 3:**
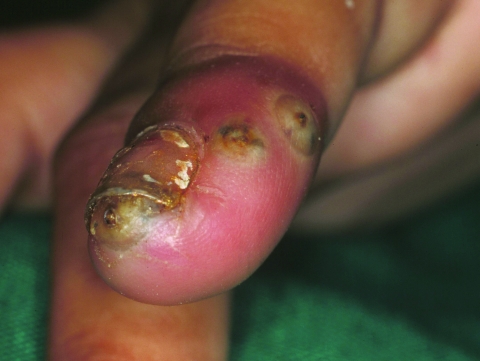
Second finger of the right hand of the same patient. Acute inflammation with intense erythema and a slight edema is shown. Two stage III lesions are located at the lateral side of the finger and another lesion is lifting up the nail.

A 6-year-old girl had 96 lesions. Of the 96, a total of 30 were located on the toes; all toes but one were infested with sand fleas; one sand flea was trying to penetrate the skin of on the second toe of the left foot (Figure 2). Forty-eight lesions occurred on the soles of the feet, and eight were located on the heels. Ten lesions were found on the hands and impeded the girl from gripping (Figure 3). Most lesions were surrounded by severe erythema and edema. Pustules and suppurations were frequent. Sleep was reported to be severely disturbed, and the child woke up in the night and cried. The family lived in a hut with sandy soil. The mother devoted most of her attention to two younger siblings. The mother was unmarried and illiterate.

### Case 3

**Figure 4:**
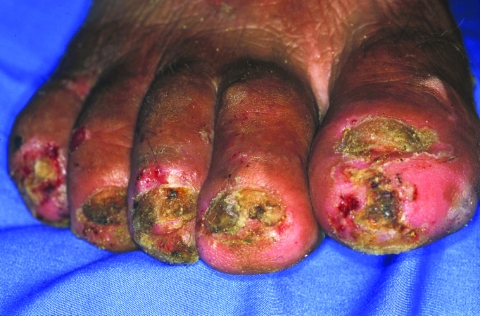
Right foot of a 50-year-old man. All nails have been lost. Embedded fleas have been manipulated by the patient, leaving innumerable sores. Desquamation and ulceration are merged. The skin tends to bleed where the stratum corneum is eroded.

A 50-year-old man had 123 lesions. Thirty-six lesions were located on the toes, 33 on the soles, and 54 on the heels. Nine nails were lost (Figure 4). Bacterial superinfection with pustules or suppuration was present on both feet. Nineteen ulcers were also found. Severe desquamation and hyperkeratosis occurred alternately. The patient had persistent pain and could walk only with considerable difficulty. He had manipulated many lesions with a nonsterile needle or a thorn and had also treated his feet with candle wax diluted in used motor oil to get rid of the sand fleas. Presumably, the self-treatment added to the aspect of general inflammation of both feet. The patient was unemployed and lingered around in the *favela* all day. He had no shoes and only wore slippers.

### Case 4

**Figure 5:**
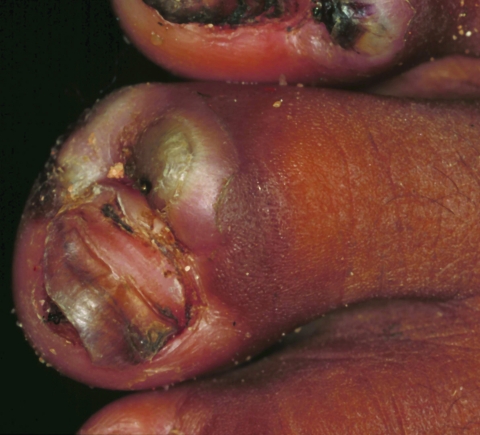
Fourth toe of a 50-year-old women. The nail is lifted up by a lesion. An abcess has formed near the nail wall, and the toe is distorted because of intense edema.

A 55-year-old woman had 76 lesions. Forty-eight lesions were located at periungual sites, and 17 in other regions of the toes. Eleven lesions were on the soles of the feet. Both feet were edematous; the edema extended over the entire lower legs. Several toes were deformed and all nails were damaged (Figure 5). Pustules and suppuration occurred in all toes. The patient was unable to walk and had to remain in her hammock. The patient was farsighted and did not have appropriate glasses. She lived alone in a small hut. The sandy compound was littered with waste and organic material.

## Discussion

With a length of <1 mm, *Tunga penetrans* is the smallest known flea species (*27*). Once burrowed in the skin of its host, the flea becomes hypertrophic, produces and releases eggs, and eventually dies. Microbiologically, the embedded flea behaves as a foreign body with a continuously enlarging surface (*8*). The parasite remains buried in the epidermis, except for the posterior parts of the abdomen bearing the anus, the genital opening, and four pairs of large stigmata. The protruding hindquarters create a sore, connecting the surface of the skin to the epidermis and, through the proboscis of the flea, to the dermis. When the skin’s surface is linked by means of a foreign body to underlying tissue layers, translocation of the normal microflora occurs, leading to a bacterial infection in the epidermis and presumably to the formation of a biofilm on the surface of the foreign body (*28*). Thus, in the vertebrate host, the infection with *T. penetrans* is a self-limiting process, and the risk for infectious complications is obvious.

In fact, older literature abounds with observations on severe pathologic findings associated with tungiasis and mentions debilitating sequelae such as phagedenic ulcers, gangrene, auto-amputations of digits, and the loss of entire limbs (*19*,*29*,*30*). Death resulting from infection with *Clostridium tetani* is not uncommon in nonvaccinated persons (*19*–*22*). Such severe sequelae of tungiasis were not only observed in native South American populations by European travelers (*2*,*15*,*31*) but were also reported by military physicians responsible for local forces in West and East Africa (*15*,*16*,*29*). In 1909, Decle (*29*) gave a vivid description of the havoc wrought by the ectoparasite on military personnel in Kenya: “At Ford Raymond the garrison consisted of 160 askaris (soldiers) and 70 porters; out of this men 72 soldiers and 30 porters were absolutely unfit for service through ulcers brought on by jiggers, and 30 more were lame.” In another garrison, Fort Grant, severe tungiasis caused illness in 50% of the local soldiers (*29*). During military operations in Cameroon in the first quarter of the 19th century, 5% of all septic admissions were due to complications of the ectoparasitosis (*15*). These accounts show that severe tungiasis was an important health problem in the underdeveloped rural hinterlands of South America and Africa until the middle of the 20th century.

In the last 50 years, severe tungiasis seems to have disappeared as a disease entity. In current textbooks the ectoparasitosis is mainly mentioned as an exotic nuisance for the casual traveler in South America and sub-Saharan Africa (*6*). Tourists seldom have more than one lesion and usually seek medical advice within 1 week of flea penetration, resulting in the extraction of the parasite at an early stage (*12*). However, we observed persons living in a underdeveloped neighborhood in Fortaleza, northeast Brazil, where patients carried up to 145 sand fleas in all stages of development.

As parasites tend to accumulate at certain predilection sites, the pathologic findings should be particularly severe in these sites. In our patients and others in the community, many infected persons have lesions in the periungual region of the toes (*9*), which explains the high frequency (69%) of nail deformation or nail loss. Furthermore, as the sole of the feet and the heel were other predilection sites, difficulty in walking was also very common.

Various mechanisms exist by which embedded fleas could induce pathologic alterations of the skin in an early stage of development. Acute inflammation with erythema, edema, pain, and itching is conceivably due to tissue damage induced by a metabolically highly active and continuously enlarging parasite. As with other blood-sucking insects, *T. penetrans* releases proteolytic enzymes during penetration and growth, causing an inflammatory response of the skin. In comparison with other ectoparasites that frequently reinfect humans, the immune response of the host might contribute to the intense inflammation observed soon after penetration.

As the lesion develops, bacterial superinfection almost inevitably occurs (*8*). During penetration, the flea breaks up the stratum corneum, allowing bacterial microcolonies on the skin surface to spread. In addition, pathogenic microorganisms on the outer surface of the flea may be actively carried into the epidermis (*8*). As the continuously expanding body of the flea (the volume increases by a factor of roughly 2,000) consists of rather smooth intersegmental skin and newly formed chitinous clasps, the embedded flea fulfills the requirement of a structural matrix to which microorganisms could easily adhere (*32*). In fact, scanning-electron microscopy of extracted fleas showed that pathogens such as streptococci and gram-negative rods formed a biofilm in the tiny grooves of newly built intersegmental skin as well as on the chitinous exoskeleton (Feldmeier and Meckes, unpub. data, 2002).

As the lesion itches immediately after the flea penetrates, patients usually start to scratch, which, in turn, promotes the entry of bacteria through the persistent sore in the epidermis. In fact, we invariably observed microabscesses in histologic sections of lesions only 2 days after penetration (*7*).

In many of our patients, bacterial superinfection was also the result of an inappropriate manipulation of lesions with nonsterile instruments by the patient or caregiver (Figures 4 and 5). The remarkable desquamation of the skin observed around late-stage lesions (Figure 4) has its histopathologic correlates in hyperkeratosis and parakeratosis of the stratum corneum (*7*).

Recently, *Wolbachia* species have been identified in the ovaries of *T. penetrans* (*33*). As antigens of these bacterial endosymbionts have been associated with the pathologic immune response in some filarial diseases (e.g., onchocerciasis), part of the intense immune response in tungiasis might also be evoked by *Wolbachia* antigens being released from decaying fleas (*34*). At present, a study is being undertaken to verify this assumption in experimentally infested rats.

Reports on severe tungiasis involve persons with particular risk factors, such as alcoholics or the mentally diseased, who are expected to have prolonged contact with the ground or are unable to care for themselves (*35*,*36*). Our data clearly show that severe tungiasis also occurs in persons without such risk factors who live in an impoverished community, when environmental, socioeconomic, and behavioral factors coexist and make frequent reinfection likely or impede the extraction of penetrated fleas in the early stage. Recently, estimates show that in northeast Brazil alone, several million people who live in communities with similar environmental characteristics to those we studied are at risk of tungiasis (*2*).

Although we cannot give an accurate estimate of severe tungiasis in the general population level, 16 (17%) of 86 patients arrived at the PHCC for reasons unrelated to the ectoparasitoses but showed important sequelae, which indicates that tungiasis is frequent on the community level. This assumption has been corroborated by a study performed in south of Brazil.

Thus, whereas severe tungiasis has disappeared from the underdeveloped rural hinterland where it formerly existed, this disease should be considered as a resurgent health problem of underdeveloped urban areas, where environmental conditions favor a high attack rate and social neglect is intricately linked to poverty and inadequate healthcare behavior. At least in Brazil, the medical profession wholly neglects this ectoparasitosis, and physicians do not diagnose tungiasis during consultation unless the condition is mentioned by the patient (*25*,*37*).

Morse (*38*) has convincingly argued that a reemerging disease is rarely a purely microbiologic event but commonly has causative cofactors such as ecologic changes, changes in human demography, international travel, or breakdown of public health measures. The results of our study suggest that poverty, social neglect, and inappropriate healthcare behavior should be added to this list.
